# Purification and Characterization of a New Antifungal Compound 10-(2,2-dimethyl-cyclohexyl)-6,9-dihydroxy-4,9-dimethyl-dec-2-enoic Acid Methyl Ester from *Streptomyces hydrogenans* Strain DH16

**DOI:** 10.3389/fmicb.2016.01004

**Published:** 2016-06-29

**Authors:** Talwinder Kaur, Amarjeet Kaur, Vishal Sharma, Rajesh K. Manhas

**Affiliations:** ^1^Department of Microbiology, Guru Nanak Dev UniversityAmritsar, India; ^2^Department of Pharmaceutical Sciences, Guru Nanak Dev UniversityAmritsar, India

**Keywords:** *Streptomyces hydrogenans* DH16, antifungal compound, fungal phytopathogens, biofungicide, biocontrol

## Abstract

In agriculture, biocontrol agents have been emerged as safe alternative to chemical pesticides where *Streptomyces* spp. and their metabolites constitute a great potential for their exploration as potent agents for controlling various fungal phytopathogens. The present study reports an antifungal compound purified from *Streptomyces hydrogenans* strain DH16, a soil isolate, using silica gel chromatography and semi preparative HPLC. The compound was characterized using various spectroscopic techniques (IR, ^1^H and ^13^C NMR) and named 10-(2,2-dimethyl-cyclohexyl)-6,9-dihydroxy-4,9-dimethyl-dec-2-enoic acid methyl ester **(SH2)**. Compound **(SH2)** showed significant inhibitory activity against fungal phytopathogens and resulted in severe morphological aberrations in their structure. Minimal inhibitory and minimal fungicidal concentrations of the compound ranged from 6.25 to 25 μg/ml and 25 to 50 μg/ml, respectively. *In vivo* evaluation of the compound showed strong control efficacy against *Alternaria brassicicola*, a seed borne pathogen, on radish seeds. In comparison to mancozeb and carbendazim, the compound was more effective in controlling damping off disease. Additionally, it promoted plant growth with increased rate of seed germination, and displayed no phytotoxicity. The compound retained its antifungal activity after its exposure to temperature of 100°C and sunlight for 1 h. Furthermore, the compound **(SH2)** when tested for its biosafety was found to be non-cytotoxic, and non-mutagenic against *Salmonella typhimurium* TA98 and TA100 strains. This compound from *S. hydrogenans* strain DH16 has not been reported earlier, so this new compound can be developed as an ideal safe and superior biofungicide for the control of various fungal plant diseases.

## Introduction

Plant pathogens, especially fungi are one of the major threats to agricultural productivity of economically important crops worldwide (Cornelissen and Melchers, 1993). Chemical fungicides are used in current agricultural practices to combat these phytopathogens. However, long term application of these chemicals has resulted in severe negative impacts on environment and human health. Furthermore, their indiscriminate and repeated use has triggered the emergence of resistance in phytopathogens, due to which several important chemical fungicides have lost their efficacy against the resistant pathogens in the field (Yang et al., 2008). These limitations of chemical fungicides and increased public concern for pesticide free food highlight the discovery and development of new safer fungicides (Coloretti et al., 2007).

In recent years, control of plant diseases using microorganisms and their bioactive metabolites has drawn greater attention as better alternative to chemical fungicides. The antifungal antibiotics of microbial origin are safe, broad spectrum, less toxic to host plants, easily biodegradable in the biosphere, thus low residue levels in environment and food. Cycloheximide and streptomycin from *Streptomyces griseus* were successfully used to control fungal and bacterial diseases in plants, respectively, for the first time (Leben and Keitt, 1954). Since then, many attempts were made to explore various antibiotics from microorganisms for control of plant diseases and some *viz*. blasticidin S, polyoxin, kasugamycin, validamycin, gopalamycin, dorrigocins, geldanamycin, nigericin, fistupyrone, jiggangmycin, phenyl acetic acid, azalomycin have been developed as fungicides for agricultural use (Hochlowski et al., 1996; Kim and Hwang, 2007).

*Streptomyces* spp. hold considerable importance in biocontrol of various plant diseases caused by diverse range of plant pests. These bacteria are the largest hub for the antimicrobial agents, and approximately two third of economically important antibiotics developed for agricultural use are from *Streptomyces* spp. Their use as potent biocontrol agents against phytopathogenic fungi has been reported by various workers (Yuan and Crawford, 1995; Xiao et al., 2002; Kanini et al., 2013; Faheem et al., 2015) and is mainly due to the production of various antifungal compounds (Doumbou et al., 2001; Xiong et al., 2012; Palaniyandi et al., 2013; Nguyen et al., 2015) and cell wall degrading enzymes such as chitinases and glucanases (Gupta et al., 1995; Taechowisan et al., 2003, 2005; Quecine et al., 2008). Streptomycetes have also been used as biofungicide formulations containing live mycelium or spores and their active metabolites. For example, mycostop (containing *S. griseoviridis* K61), Actinovate and actinoiron (containing *Streptomyces lydicus* WYEC 108) and Rhizovit^R^ (*S. rimosus*) are commercial biofungicides used to control plant diseases caused by *Phytophthora* spp., *Fusarium* spp., *Pythium* spp., *Alternaria brassicicola, Botrytis* sp., and *Rhizoctonia solani* (Tahvonen and Avikainen, 1987; Marten et al., 2000).

Although different antifungal compounds from *Streptomyces* spp. have been reported but it is just the tip of the ice berg that has been explored. Therefore, in continuous demand for new bioactive metabolites for plant protection, the present study reports the purification, characterization and biological evaluation of a new antifungal compound from *S. hydrogenans*, a strong antagonist against various fungal phytopathogens. The biosafety of the compound was also evaluated using Ames Mutagenicity and MTT (3-(4,5-dimethylthiazol-2-yl)-2,5 diphenyltetrazolium bromide) cytotoxicity tests.

## Materials and Methods

### Microorganisms and Maintenance

*Streptomyces hydrogenans* strain DH16 (GenBank accession no. JX123130) was isolated from soil procured from Dalhousie (32.53° N, 75.98° E), Himachal Pradesh, India (Kaur and Manhas, 2014) and maintained on starch casein nitrate agar slants at refrigeration temperature (4°C). Twenty percent glycerol stocks were also prepared and stored at -20°C during the study. The three test phytopathogenic fungi *viz*. *Alternaria brassicicola* (MTCC 2102), *A. solani* (MTCC 2101), and *Colletotrichum acutatum* (MTCC 1037) were obtained from Microbial Type Culture Collection (MTCC), Institute of Microbial Technology (IMTECH), Chandigarh, India. *Fusarium moniliforme, Alternaria alternata* were isolated in lab. All the fungal cultures were maintained on Potato dextrose agar (PDA) slants at 4°C.

### Production of Antifungal Metabolites

Production of antifungal metabolites from *S. hydrogenans* strain DH16 was carried out according to Kaur and Manhas (2014). The actinobacterium was grown on starch casein nitrate agar medium at 28°C for 7 days and then growth was scrapped and transferred aseptically into the SCN broth to develop seed culture. After 48 h of incubation, the seed culture was inoculated into 250 ml Erlenmeyer flasks containing 50 ml of production medium containing g/L: starch, 12; soyabean meal, 2.5; K_2_HPO_4_, 1.8; NaCl, 2; Casein, 0.3; MgSO_4_, 0.05; FeSO_4_, 0.01; and CaCO_3_, 0.02. The fermentation was carried out at 28°C at 180 × *g*, and after 3 days of incubation, culture broth was centrifuged at 10000 × *g* for 20 min at 4°C to obtain cell free culture supernatant.

### Extraction and Purification of Metabolites

For the recovery of active metabolites, culture supernatant (5 l) was extracted twice with equal volume of ethyl acetate (EA). The organic phase was separated, treated with Na_2_SO_4_ and then concentrated to dryness under vacuum at 45°C using rotary evaporator (BUCHI Rota vapor R-200). For the purification of antifungal compounds, the resulting solids (1 g) re-dissolved in small volume of methanol were subjected to column chromatography using silica gel (60–120 mesh size; column, 1.0 cm × 35 cm) packed and pre-equilibrated with chloroform. The column was then eluted step-wise with linear gradients of: chloroform/methanol (100:0, 90:10, 80:20,70:30, 60:40, 50:50, 40:60, 30:70, 20:80, 10:90, and 0:100) at a flow rate of 2 ml/min. About 200 ml of each gradient was used for elution, and a total of 88 fractions of 25 ml each were collected and concentrated. Each fraction was then subjected to disk diffusion assay to determine their antifungal activity against *A. brassicicola* and fractions showing antifungal activity were pooled together and concentrated. Final purification of the active compounds was achieved by preparative RP-HPLC: Shimadzu Microsorb MV, 100 mm × 10 mm ID, 10 μm, flow rate 3 ml/min, gradient Acetonitrile:H_2_O 50% in 5 min, 50–70% in 15 min and 70–50% in 17 min, and UV detection at 210 nm. All the peaks of chromatogram were collected using a fraction collector coupled with the HPLC system, then concentrated and screened for antifungal activity. Three peaks which showed activity were rechromatographed with the same solvent system to check the purity to homogeneity level.

### Characterization of Active Peak 2

The peak **2** was characterized based on various physicochemical and spectroscopic properties. Appearance, color, odor, and solubility were determined according to the standard procedures. The UV-Visible spectrum was recorded qualitatively on UV-Visible Spectrophotometer (Shimadzu) in the range of 200–400 nm using acetonitrile as reference solvent.^1^H NMR and ^13^C NMR spectra were recorded in chloroform-d [99.8 atom% D, containing 0.1% (v/v) tetramethylsilane (TMS)] at 25°C on 500 MHz AVANCE III Bruker spectrometer equipped with a 5 mm double channel solution state probe. The chemical shifts are reported in parts per million (ppm) relative to TMS (δ0.0) used as internal standard. Mass spectrum (HR-MS) was recorded with Bruker MICROTOF II spectrometer. IR spectrum was recorded with Perkin–Elmer FTIR-C92035 Fourier-Transform spectrophotometer in the range 400–4000 cm^-1^ by using CHCl_3_ as the medium for the preparation of the samples.

### Antifungal Activity of Purified Compound

The antifungal activity of the purified compound SH2 was tested against *A. brassicicola, A. solani* and *C. acutatum, F. moniliforme and A. alternata* causing various diseases on diverse host plants. The activity was determined in terms of zone of inhibition by using Kirby–Bauer well diffusion assay (Bauer et al., 1996). Wells of 4 mm diameter were made on PDA plates seeded with the test fungal pathogens. Then aqueous solutions of purified compound, cycloheximide and chemical control agents (carbendazim and mancozeb) were made at concentration of 1 mg/ml and 100 μl of each were added into wells. The diameters of the resultant zones of inhibition were measured in mm after 48–72 h of incubation. Each experiment was performed in duplicates and repeated thrice.

### Effect of Purified Compound on Fungal Morphology

The effect of purified compound **(SH2)** on morphology of *A. brassicicola* and *F. moniliformae* was studied microscopically. Mycelia of *A. brassicicola* and *F. moniliforme* were taken from periphery of the inhibition zones around the well (containing **SH2**) and from control plate and placed on glass slide in a drop of sterile water. The coverslip was placed on the film and then visualized under bright field microscope at 40× (Olympus). Microphotographs were taken using a digital camera.

### Minimal Inhibitory Concentration (MIC) and Minimal Fungicidal Concentration (MFC)

Minimal inhibitory concentration of EA extract was worked out by 96 well microtitre plate method (Díaz-Dellavalle et al., 2011) using different concentrations (12.5, 25, 50, 100, 250, 500, and 1000 μg/ml) of extract in EA. The spore suspension of test fungus was prepared by scrapping the spores from 5-day-old PDA culture plate with fresh PDB. In 96 well microplates, 100 μl of fungal spore suspension (1 × 10^5^ spores/ml) was mixed with 100 μl of extract of different concentrations. Control well contained 100 μl of fungal spore suspension and 100 μl of EA. Control blanks consisted of 100 μl of extract of different concentrations with 100 μl of PDB and other contained 100 μl of PDB and 100 of EA only. The microplates were incubated at 28°C and readings were taken with microplate reader at 595 nm after 48 h. MIC values were calculated by comparing the growth in wells containing extract to the growth in control wells and is the lowest concentration that resulted in 80% inhibition in growth compared to the growth in control well. Further MFC was determined by plating 20 μl of the broth from each well on fresh PDA plates. The plates were then incubated at 28°C until growth was visible in the control subculture. The MFC will be that lowest concentration where no visible growth will be observed on plate. The experiments were performed in triplicates. Similarly, the MIC and MFC values for purified compound were determined using different concentrations (6.25, 12.5, 25, 50, 100, and 200 μg/ml).

### Biocontrol of *A. brassicicola* on *Raphanus sativus* Seeds by Purified Compound

*In vitro* and *in vivo* experiments were used to evaluate the biocontrol potential of compound to control *A. brassicicola* on surface sterilized radish seeds. The sterilized seeds were first artificially infested with the pathogen by immersing them for 4 h in spore suspension, prepared from 5-day-old *A. brassicicola*, in presence of 1% carboxy methyl cellulose (CMC; 10^5^–10^7^ spores/ml). The pathogen infested seeds were then soaked in 1 mg/ml solution of compound and chemical control agents. After 1 h, all seeds were dried in laminar flow on a sterile filter paper and used for further experiments. Each treatment consisted of three replicates with 10 seeds each.

#### *In Vitro* Blotter Test

The moistened blotters were first used to determine the effect of compound to reduce damping off due to seed-borne *A. brassicicola* on radish plants grown from artificially infected seeds. Three replicates of 10 seeds per treatment were placed in Petri dishes (10 seeds per plate) already lined with moist filter paper and covered loosely with another filter paper. The number of germinated seeds and healthy and diseased seedlings were recorded after 7 days of incubation at 28°C in the dark. Seedling vigor (V) was determined by measuring root and shoot lengths of 10 seedlings (selected randomly) and was calculated according to the equation:

$$V=(L_{s}{+L}_{r})\times G$$

Where L_s_ is average shoot length in mm and L_r_ is average root length in mm and G is % germination (Andresen et al., 2015).

#### *In Vivo* Pot Experiment

Treated seeds were sown in pots containing sterilized soil with 10 seeds per pot. The pots were kept under natural conditions and were watered daily. Seed germination, emergence of healthy seedlings, mean fresh, and dry weights of emerged plants and seedling vigor were recorded after 15 days of sowing. To determine the dry weight of plants, harvested plants were placed separately on filter papers in oven at 60°C for 48 h and then weighed using weighing balance.

### Stability of Compound SH2

To determine heat stability, compound **SH2** was heated at 37, 50, 70, and 100°C for 1 h. Photostability was also tested by exposing the compound separately to sunlight for 1 h. All the treated samples were then checked for residual activity with respect to untreated control against *C. acutatum.*

### Safety Evaluation

Purified compound **(SH2)** was evaluated for toxicity testing *viz*. phytotoxicity, mutagenicity, and cytotoxicity.

#### Phytotoxicity Testing

Phytotoxicity was checked by treating sterilized seeds with purified compound. In control, the compound was replaced with water. Treated seeds were then sown in sterilized soil and data for important agronomic parameters (seed germination, seedling vigor and weight of plants) were recorded after 10 days.

#### Mutagenicity Studies

The mutagenicity of purified compound was determined using *Salmonella* histidine point mutation assay proposed by Maron and Ames (1983) with slight modifications suggested by Bala and Grover (1989). For toxicity testing, 0.1 ml of bacterial culture and 0.1 ml of compound **SH2** at different concentrations (50, 100, and 250 μg/plate) was added to 2 ml of top agar and poured onto the minimal agar plates followed by incubation at 37°C for 48 h. To determine the spontaneous reversion which is characteristic of the tester strains (TA98 and TA100), negative control (0.1 ml bacterial culture + 0.1 ml DMSO per plate) was run while 4-Nitro-*o*-phenylenediamine (20 μg/0.1 ml/plate) and sodium azide (2.5 μg/0.1 ml/plate) were used as a positive control mutagens for strains TA98 and TA100, respectively. After incubation for 48 h, the number of revertant his^+^ bacteria colonies were scored. The mutagenic potential of the purified compound was determined by comparing the number of colonies with control plates where no test compound as well as mutagen was added.

#### *In Vitro* Cytotoxicity

The MTT assay was used for determining *in vitro* cytotoxicity following the method given by Mosman (1983) using Chinese Hamster Ovary (CHO), a non-tumor or normal cell line obtained from National Centre for Cell Science (NCCS), Pune (India). Cells of CHO were grown in tissue culture flask in complete growth medium [Roswell Park Memorial Institute (RPMI)- 1640 medium with 2 mM glutamine, 100 units ml-1 streptomycin and supplemented with 10% Foetal Calf Serum (FCS) and 100 units/ml penicillin] at 37°C in an atmosphere of 5% CO_2_ and 90% relative humidity. The cells at subconfluent stage were harvested from the flask by treatment with trypsin (0.05% in PBS containing 0.02% EDTA) for determination of cytotoxicity. Cells with viability of more than 98%, as determined by trypan blue exclusion, were used for assay. The cell suspension of 1 × 10^5^ cells/ml was prepared in complete growth medium for determination of cytotoxicity. Stock solution of purified compound (100 μg/ml) was prepared in sterile filtered DMSO. The compound was serially diluted with complete growth medium containing 50 μg/ml of gentamicin to obtain working test solutions of different concentrations.

The 100 μl of cell suspension (1 × 10^5^ cells/ml) were seeded in 96-well tissue culture plates (Microtest^TM^, Falcon, USA), followed by addition of 100 μl of each concentration (30, 50, and 100 μg/ml) of **SH2**, after 24 h incubation. Respective control cells were treated with medium only. After 42 h of treatment the spent medium was discarded by inverting the plate on a tissue towel and 100 μl of MTT prepared in non-serum medium (0.5 mg/ml; without FCS) was added to each well. After 2 h of incubation, medium was discarded and blue formazan crystals formed by MTT reaction were dissolved in 100 μl of dimethyl sulfoxide (DMSO, Emplura^®^, Millipore) in each well. The color was read at 540 nm in the Elisa plate reader (Multiscan^®^ EX by Labsystems, Finland). The proliferation of cells under treatment was assessed according to following formula:

$$\mathrm{Percentage}\;\mathrm{of}\;\mathrm{proliferation}=\frac{\mathrm{Absorbance}\;\mathrm{of}\;\mathrm{test}}{\mathrm{Absorbance}\;\mathrm{of}\;\mathrm{control}}\times 100$$

% Growth inhibition = 100 – % Cell growth

### Statistical Analysis

All the experiments were repeated twice and the data (expressed as the mean ± SD) obtained from these experiments were subjected to statistical analysis. Tukey’s *post hoc* test was done with the help of ASSISTAT (7.7 beta) to compare the means.

## Results

### Isolation and Purification of the Compound

**FIGURE 1:**
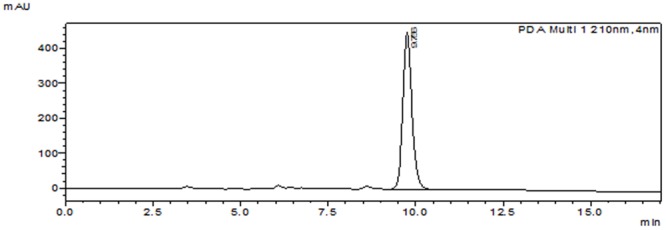
**Purified peak of compound SH2**.

The streptomycete DH16 was grown in SCN broth for the production of active antifungal metabolites. After third day of incubation, broth was centrifuged, extracted, and concentrated to yield dark brown residue which was subjected to silica gel column chromatography for isolation of active compounds. Three active fractions (no. 9–11) eluted with chloroform:methanol (90:10) in silica gel chromatography showed antifungal activity and then pooled together based on their similar TLC pattern and concentrated. The pooled fraction was then further fractionated on semi-preparative HPLC and individual peaks were collected. The peak with retention time of 9.61 min showed antifungal activity against the test pathogenic fungus (*A. brassicicola*). To check the homogeneity of active compound, collected peak was chromatographed under similar conditions and single peak was obtained which indicated the purity of the compound (**Figure 1**).

### Physical Properties of the Purified Compound

The compound was light yellowish in color and was odorless. It was soluble in most of the organic solvents but was sparingly soluble in water.

### Chemical Characteristics of the Purified Compound (SH2) from *S. hydrogenans* DH16

The compound responsible for antifungal activity was characterized by various spectroscopic techniques such as IR, ^1^H and ^13^C NMR spectra, and mass spectroscopy. **IR** (CHCl_3_): ν_max_ (cm^-1^) = 3441, 3054, 2986, 2685, 2305, 1754, 1606, 1422, 1267, 1151, 908, 896, 752; **^1^H NMR** (500 MHz, CDCl_3_): δ = 7.45 (d, 1H, *J* = 5.5 Hz, C2-H), 6.13 (distorted d, 1H, *J* = 4.2 Hz, C3-H), 5.06–5.03 (m, 1H, C6-H), 3.66 (dist. s, 3H, OCH_3_), 1.80–1.77 (m, 4H), 1.70–1.65 (m, 4H), 1.52–1.37 (m, 8H), 1.17–1.14 (m, 8H, 2x CH_3_, C7-H), 0.92–0.85 (m, 8H,); **^13^C NMR** (150 MHz, CDCl_3_): δ = 173.1 (C = O), 156.2 (C-2), 121.5 (C-3), 83.3, 72.8, 71.7, 41.1, 39.9, 34.2, 33.1, 32.3, 29.8, 29.6, 26.3, 24.9, 23.5, 14.6, 8.2; **MS** (TOF, ESI): *m*/*z*:calculated for C_21_H_38_O_4_: 354.2; found: 377.1 [M+ Na]^+^ (Supplementary Figures 1–5).

**FIGURE 2:**
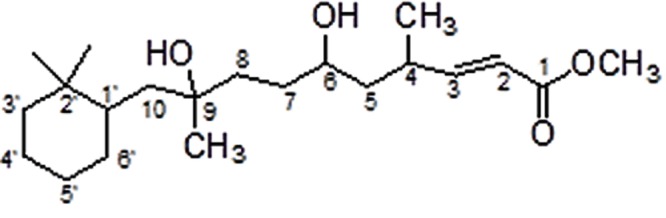
**Proposed structure of purified compound SH2**.

In the proton spectrum proton of C-2 appeared as doublet at δ 7.45 with *J* = 5.5 Hz and proton of C-3 appeared as distorted doublet at δ 6.13 with *J* = 4.2 Hz. Proton of remaining carbons in the compound showed multiple resonances by two bond and three bond couplings. ^13^C NMR of the compound showed carbonyl resonance at δ 173.1 which is further revealed by IR spectrum showed band at 1754 cm^-1^. Carbon NMR also showed resonances of two olefinic carbons at 156.2 (C-2) and 121.5 (C-3), respectively. All aliphatic carbon resonances of compound also appeared in ^13^C NMR below 100 ppm. The alcoholic function is confirmed by IR spectrum band at 3441 cm^-1^ and also reveals the other stretching and bending vibrations of functionalities in compound; its mass spectrum showed the molecular ion peak at m/z 377.1 (M+ Na)^+^, which corresponds to the molecular formula of compound. On the basis of these observations, the purified compound is proposed to be 10-(2,2-Dimethyl-cyclohexyl)-6,9-dihydroxy-4,9-dimethyl-dec-2-enoic acid methyl ester (**Figure 2**).

### Antifungal Activity of Compound (SH2)

**Table 1** depicts the antifungal spectrum of purified compound at concentration of 100 μg against various fungal pathogens. The results showed that it significantly inhibited the test fungi with inhibition zones in the range of 15–40 mm as against 15–32 mm zones resulted from carbendazim and mancozeb. In case of *C. acutatum* and *Alternaria* spp., the purified compound was more effective as compared to chemical control agents. In addition to antifungal activity, the purified compound also showed inhibitory activity against *Candida albicans* and *Bacillus subtilis* (data not shown).

**Table 1:** Antifungal activity of purified compound SH2 and chemical control agents when used at a concentration of 100 μg.

Test fungus	*Zone of inhibition (mm)*		
	Purified Compound	Mancozeb	Carbendazim
*A. brassicicola*	35 ± 0.1a	16 ± 0.5b	–
*A. solani*	30 ± 0.5a	17 ± 0.0c	–
*C. acutatum*	40 ± 1.0a	19 ± 0.2b	32 ± 0.0c
*F. moniliformae*	25 ± 0.5a	15 ± 0.0c	22 ± 0.5b
*A. alternata*	35 ± 1.0a	15 ± 0.5b	–
*A. mali*	32 ± 1.0a	15 ± 0.1b	–

*Means followed by different letters with in a row are significantly different; Tukeys HSD, *p* ≤ 0.05).*

### Antifungal Effects of Purified Compound

**FIGURE 3:**
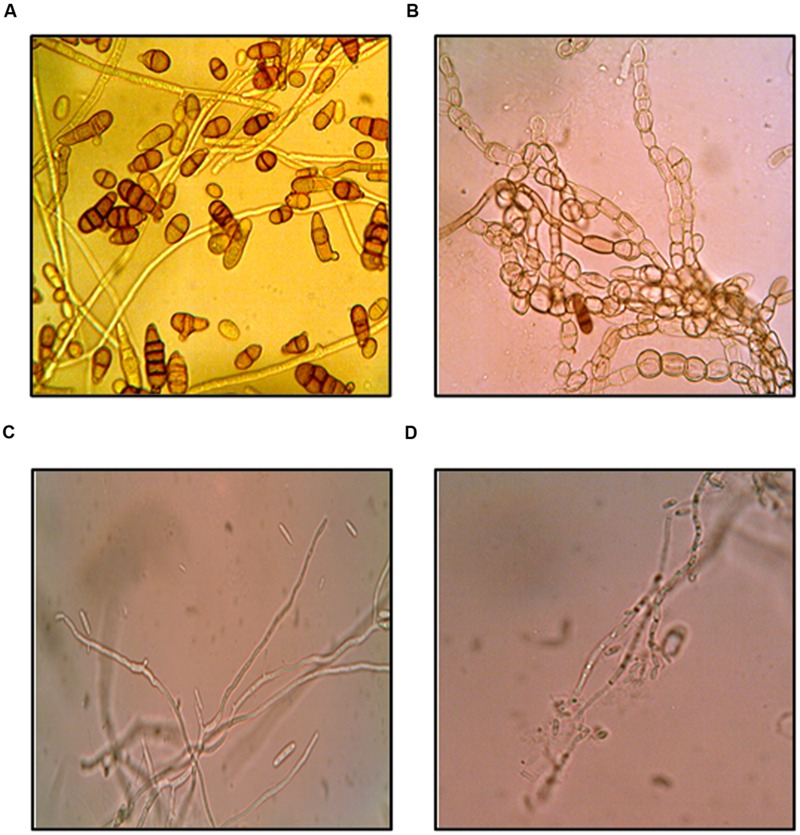
**Antifungal effects of purified compound **SH2** on *A. brassicicola***(A)** control **(B)** treated; and *F. moniliforme***(C)** control **(D)** treated**.

The effect of purified compound on spore germination and hyphal morphology was studied for *A. brassicicola* and *F. moniliforme.* Microscopic observations showed that purified compound significantly inhibited spore germination in both the fungal pathogens as compared to control (*p* < 0.05). Severe morphological abnormalities such as hyphal swellings resulted in bulbous structures, thinning of hyphae, discoloration of hyphae were also observed. Additionally, purified compound also resulted in pigmentation loss in mycelial structure of *A. brassicicola* (**Figure 3**). MIC and MFC values of the purified compound and EA extract of streptomycete were determined by 96 well plate method and are shown in **Table 2**. The crude extract showed significant antifungal activity against all the tested fungi with MIC values of 50, 25, 100, 50, and 250 μg/ml for *A. brassicicola, C. acutatum, A. solani, A. alternata*, and *F. moniliforme*, respectively. The MIC and MFC values of purified compound were 25 and 50 μg/ml for *A. brassicicola* and were 6.25 and 25 μg/ml for *C. acutatum.*

**Table 2:** MIC and MFC values of crude extract and purified compound SH2 against phytopathogenic fungi.

Test fungus	Crude extract		Purified Compound	
	MIC	MFC	MIC	MFC
*A. brassicicola*	50	250	25	50
*A. solani*	100	500	ND^a^	ND
*C. acutatum*	25	75	6.25	25
*F. moniliforme*	250	500	100	200
*A. alternata*	50	250	ND	ND

*^a^ND, not determined.*

### Biocontrol of *A. brassicicola*

The results of *in vitro* as well as *in vivo* experiments showed ability of compound to control *A. brassicicola* (**Table 3**). In comparison to chemical control agents, the compound purified from *S. hydrogenans* strain DH16 was found to be more potent causing significant inhibition of pathogen on seeds and resulting in emergence of healthy seedlings. In *in vivo* experiments, no germination was observed in seeds, treated with pathogen only. Treatment of pathogen infested seeds with compound improved seed germination and seedling vigor to 71.4% and 1890, respectively, and are comparable to control. On the other hand delayed seed germination of 42 and 14.2% was observed in case of mancozeb and carbendazim treated seeds, respectively. The percentage of healthy seedlings and their fresh and dry weights were also significantly higher in case of seeds treated with compound (*p* ≤ 0.05).

**Table 3:** *In vitro* and *in vivo* protective effect of purified compound SH2 to control *A. brassicicola* on seeds of *R. sativus.*

	*In vitro*					*In vivo*				
Seed treatment	Seed germination (%)	Healthy seedlings (%)	^a^Fresh weight of seedling (g)	Dry weight of seedling (g)	Seedling vigor	Seed germination (%)	Healthy seedlings (%)	^a^Fresh weight of seedling (g)	Dry weight of seedling (g)	Seedling vigor
Pathogen only	13.3 ± 2.8a	0	0.010 ± 0.01a	0.001 ± 0.01a	16.95 ± 3.7a	10.0	0	0.0	0.0	0.0
Pathogen and mancozeb	50.0 ± 4.0b	46.6 ± 4.8a	0.12 ± 0.05b	0.01 ± 0.02b	567.45 ± 12.2b	60 ± 8.1a	55.5 ± 2.3a	0.16 ± 0.02a	0.015 ± 0.001a	462 ± 5.3a
Pathogen and carbendazim	33.3 ± 3.0c	30.0 ± 2.0b	0.09 ± 0.02c	0.005 ± 0.01a	347 ± 4.9c	20.0 ± 5.0b	33.3 ± 1.0b	0.12 ± 0.05b	0.010 ± 0.002b	113.6 ± 1.7b
Pathogen and compound	80.0 ± 2.5d	83.3 ± 2.5c	0.27 ± 0.05d	0.030 ± 0.001c	1890 ± 5.0d	66.6 ± 0.5c	75.0 ± 2.0c	0.28 ± 0.04c	0.03 ± 0.008c	1264 ± 0.5c
Water	93.3 ± 1.8e	100 ± 0.0d	0.29 ± .07d	0.035 ± 0.007cd	1978 ± 1.5e	83.33 ± 1.6d	100 ± 0.0d	0.34 ± 0.05d	0.039 ± 0.005d	1440 ± 2.3d
Compound SH2 only	100 ± 0.0f	100 ± 0.0d	0.31 ± 0.09d	0.04 ± 0.008d	2350 ± 3.2f	93.3 ± 0.17e	100 ± 0.0d	0.40 ± 0.02e	0.047 ± 0.007e	1805 ± 2.8e

*Means followed by different letters with in a column are significantly different; Tukeys HSD, *p* ≤ 0.05; ^a^Average weight per seedling.*

### Stability of Compound

No loss in antifungal activity of compound was observed after its exposure to temperatures up to 70°C. However, a decrease of 12.5 and 37.5% in the residual activity was observed after boiling (100°C for 1 h) and autoclaving (121°C for 30 min), respectively. The compound was also found to be photostable as only 5% loss was observed in activity against *C. acutatum*.

### Toxicity of Compound

**FIGURE 4:**
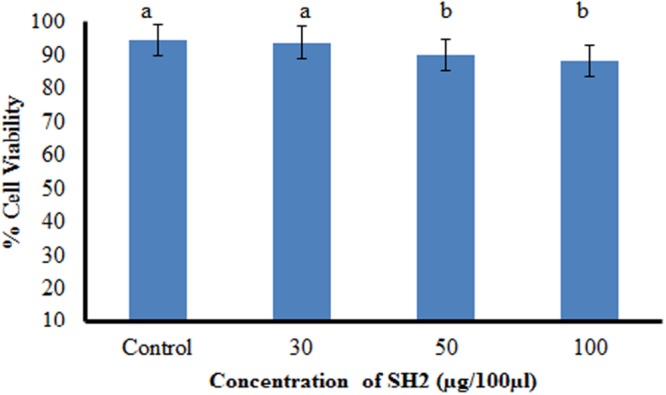
**Cytotoxicity of compound SH2 against CHO cell line as determined by MTT assay; bars with different letters are statistically different (Tukey’s HSD, *p* ≤ 0.01)**.

To work out the biosafety of the purified compound, phytotoxicity, Ames mutagenicity and MTT cytotoxicity tests were carried out. The compound was found to be non-phytotoxic because the seedlings emerged from seeds treated with compound showed increase in all the growth traits (shoot length, root length, seedling vigor). Rather than showing any phytotoxicity, **SH2** triggered as well as enhanced seed germination as compared to water treated seeds. The emerged seedlings were also found to be healthier than the control plants as shown by higher seedling weights. Antifungal compound (**SH2**) was also non-mutagenic at all the concentrations used in the experiment. The numbers of revertant colonies in both the positive controls were numerous; whereas, the numbers colonies of bacteria in the presence of the purified compound were similar to that of spontaneous revertant colonies for TA98 and TA100 (**Table 4**). Further, the compound showed insignificant cytotoxicity, i.e., only 11.6% inhibition (or 88.8% viable cells) against CHO cell line at the highest tested concentration (**Figure 4**).

**Table 4:** Revertant mutants of TA98 and TA100 *S. typhimurium* after treatment with various doses of purified antifungal compound SH2 of *S. hydrogenans* DH16.

Treatment	Dose (μg/plate)	No. of colonies	
		TA98^a^	TA100^a^
Spontaneous^b^		24.33 ± 2.0a	187.33 ± 11.6a
Positive Control^c^		962.33 ± 4.5b	
(NPD)	20	
(Sodium azide)	2.5		1103.67 ± 18.2b
Negative control^d^		25.0 ± 1.5a	185.5 ± 0.5a
(DMSO)
Purified	50	26.0 ± 0.5a	185.0 ± 1.5a
compound SH2^e^	100	25.33 ± 1.0a	185.6 ± 5.0a
	250	25.6 ± 3.5a	187.0 ± 3.9a

*^a^Values are Means ± SD (*n* = 3) followed by different letters with in a column are significantly different; Tukeys HSD, *p* ≤ 0.05; ^b^Mean number of spontaneous revertants as determined in assays without **SH2** and standard mutagens; ^c^Mean number of revertants induced by reference mutagens, sodium azide and 4-Nitro-*o*-phenylenediamine (NPD); ^d^Mean number of revertants induced by DMSO in the absence of extract and mutagen; ^e^Mean number of revertants induced by **SH2** at different concentrations.*

## Discussion

*Streptomyces* species produce vast array of antifungal compounds which play important role in biocontrol of various fungal plant diseases. These bacteria constitute major portion of total antibiotics used in agricultural sector and are still great reservoirs of new antibiotics (Tanaka and Omura, 1993). This study further adds to the potential of *Streptomyces* spp. as unexhausted source of potent antifungal compounds which can be exploited as biofungicides for agricultural use.

Here in this work, purification of compound from the culture extracts of *S. hydrogenans* strain DH16 using various chromato graphic techniques resulted in apparently new antifungal compound with broad spectrum activity against different phytopathogenic fungi. This compound is proposed to be 10-(2,2-Dimethyl-cyclohexyl)-6,9-dihydroxy-4,9-dimethyl-dec-2-enoic acid methyl ester on the basis of various spectroscopic techniques.

The importance of the study lies in the fact that it is the first report on production of new potential antifungal compound by this species. The purified compound (**SH2)** showed more activity against phytopathogenic fungi esp. *Alternaria* spp. and *Colletotrichum* spp. as compared to cycloheximide and chemical fungicides (carbendazim and mancozeb). Carbendazim exhibited no activity and mancozeb showed weak activity against all the tested *Alternaria* spp. Similarly, indole-3-carboxylic acid, another bioactive compound from *Streptomyces* sp. TK-VL_333 showed better activity than that of mancozeb whereas less activity than carbendazim when tested against *F. oxysporum* (a wilt pathogen; Kavitha et al. (2010). However, compound **SH2** isolated in the present study was found to be superior to both the fungicides in terms of activity against *Fusarium* sp. also.

The low MIC and MFC values of purified compound which varied from 6.25 to 200 μg/ml depending upon the sensitivity of test fungi further demonstrated its effectiveness to control the fungal plant pathogens. It showed lowest MIC value (6.25 μg/ml) against *C. acutatum* and *C. gloeosporioides* and highest value (100 μg/ml) against *F. moniliforme*. In contrast, the MIC value of 3-methylcarbazole produced by *Streptomyces* LJK109 against *C. gloeosporioides* was found to be high (30 μg/ml; Taechowisan et al., 2012). Hwang et al., 2001 reported the MIC values in the range of 50 to >1000 μg/ml of compounds, phenylacetic acid, and sodium phenylacetate isolated from *Streptomyces humidus* strain S5-55 which are again higher than the compound **SH2** of present study.

The biocontrol potential of purified compounds obtained from *Streptomyces* spp. in controlling different fungal phytopathogens and reducing their disease incidences in *in planta* experiments has been reported (Matsuda et al., 1998; Hwang et al., 2001; Bordoloi et al., 2002; Kavitha et al., 2010). In current study also, the *in vitro* and *in vivo* experiments showed that the compound effectively controlled the development of seed borne damping off of radish seedlings caused by *A. brassicicola* when used at the concentration of 1 mg/ml whereas the mancozeb showed less control efficacy against the disease at the same concentration. Carbendazim was found to be least effective. In the absence of pathogen, the seedlings emerged from compound treated seeds were found to be more healthier than the seedlings in control. Similarly, SPM5C-1 from *Streptomyces* sp. PM5 when applied at 500 and 250 μg/ml significantly suppressed the sheath blight disease in rice and also increased the growth parameters as compared to the control in the absence of the pathogen (Prabavathy et al., 2006).

For commercial application of biologically active compounds (to be used as biopesticides/plant growth promoting agent) in agriculture sector, it is important to determine their phytotoxicity. Cycloheximide (isolated from *S. griseus*), the first compound used to control fungal and bacterial diseases in plants, showed phytotoxicity. Therefore, use of cycloheximide as an agent for plant disease control is restrained because of its toxicity to the host plants (Ford et al., 1958). The application of carbendazim, a systemic fungicide showed phytotoxicity by negatively affecting the plant biomass in *Nicotiana tabacum* (García et al., 2003). Walia et al. (2014) demonstrated the deleterious impact of mancozeb on soil microflora, nitrification, ammonification, carbon mineralization, soil enzymes, and soil microbial biomass which in turn may result in harmful effects on nutrient uptake and plant growth. However, the antifungal compound purified in present work did not show any phytotoxicity in both *in vitro* and *in vivo* experiments. Rather, it enhanced the rate of seed germination and seedling vigor in radish seedlings compared to control plants and therefore can also be used for enhancing plant growth in addition to controlling the pathogens. This data further suggests the superiority of compound **SH2** over chemical fungicides both in terms of activity as well as phytotoxicity.

The ability to tolerate various factors (light, temperature, and pH) in natural environment is very crucial for any agro active compound. Therefore, for commercial application, compound should be thermostable, photostable, and pH stable. The compound obtained from *S. hydrogenans* strain DH16 was found to be highly thermo and photostable. Prapagdee et al. (2008) and Jayaprakashvel et al. (2010) also reported considerable thermo and photostability of antifungal compounds produced by *S. hygroscopicus* and *Trichothecium roseum* MML00l, respectively.

Further toxicity of the compound was tested by Ames mutagenicity test and *in vitro* cytotoxicity test. Ames test is useful in correlating *in vitro* bacterial mutagenesis with *in vivo* carcinogenicity in animals because positive Ames test indicates that the tested chemical is mutagenic and therefore may act as a carcinogen, because cancer is often linked to a mutation. The purified compound **SH2** obtained from *S. hydrogenans* strain DH16 was found to be potentially bio safe as it did not show any mutagenic response (at all tested concentrations) against *S. typhimurium* strains TA98 and TA100 in Ames mutagenicity test. The present study gets further credence as the results of MTT assay revealed **SH2** to be non-cytotoxic as 88.8% viable cells of CHO cell line were observed in its presence at the highest tested concentration.

## Conclusion

This study demonstrates the purification and characterization of a new heat and photo stable antifungal compound, with plant growth promoting potential, from *S. hydrogenans* strain DH16, showing more promising activity against a variety of fungal phytopathogens as compared to standard chemical fungicides. The non-phytotoxic, non-mutagenic, and non-cytotoxic nature of the compound suggests that it might serve as a new, safe, and broad spectrum biofungicide to combat serious plant diseases. Therefore, the compound **SH2** can be developed as a better replacement to chemical fungicides as effective plant chemotherapeutic agent.

## Author Contributions

TK was involved in the planning and execution of the research work; analysis and interpretation of the data; manuscript writing following the suggestions of the research supervisor. VS analyzed, interpreted and characterized the compound on the basis of different spectroscopic techniques and drafted related content of the manuscript. AK provided fungal cultures *Fusarium moniliforme, Alternaria alternata*, and *Alternaria mali*; helped in analysing the data and editing of the manuscript. RM as research supervisor of TK was involved in the design and planning of research work; analysis and interpretation of data; drafting as well as critical editing of the manuscript for intellectual subject matter. All authors approved the final version of the manuscript for publication and agreed to be accountable for all aspects of the work in ensuring that questions related to the accuracy or integrity of any part of the work are appropriately investigated and resolved.

## Conflict of Interest Statement

The authors declare that the research was conducted in the absence of any commercial or financial relationships that could be construed as a potential conflict of interest.
