# Supplementary material for: Purification and Characterization of a New Antifungal Compound 10-(2,2-dimethyl-cyclohexyl)-6,9-dihydroxy-4,9-dimethyl-dec-2-enoic Acid Methyl Ester from Streptomyces hydrogenans Strain DH16
Source: Front Microbiol. 2016 Jun 29;7:1004. doi: 10.3389/fmicb.2016.01004 (PMC4926525; doi:10.3389/fmicb.2016.01004)
Supplement: Supplementary file 1 [file Presentation_1.ZIP › presentation 1.pptx]

## Slide 1
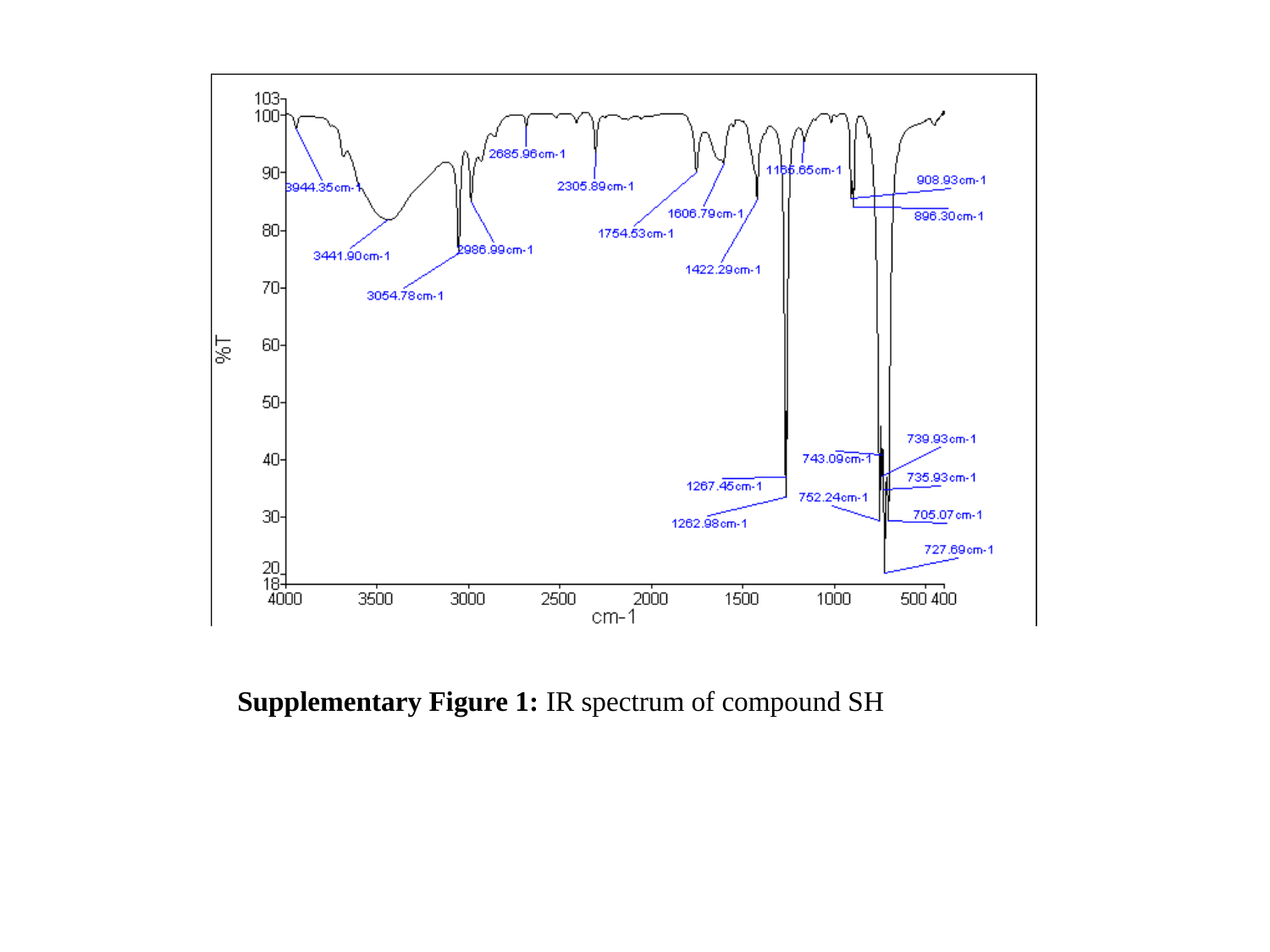

Supplementary Figure 1: IR spectrum of compound SH

## Slide 2
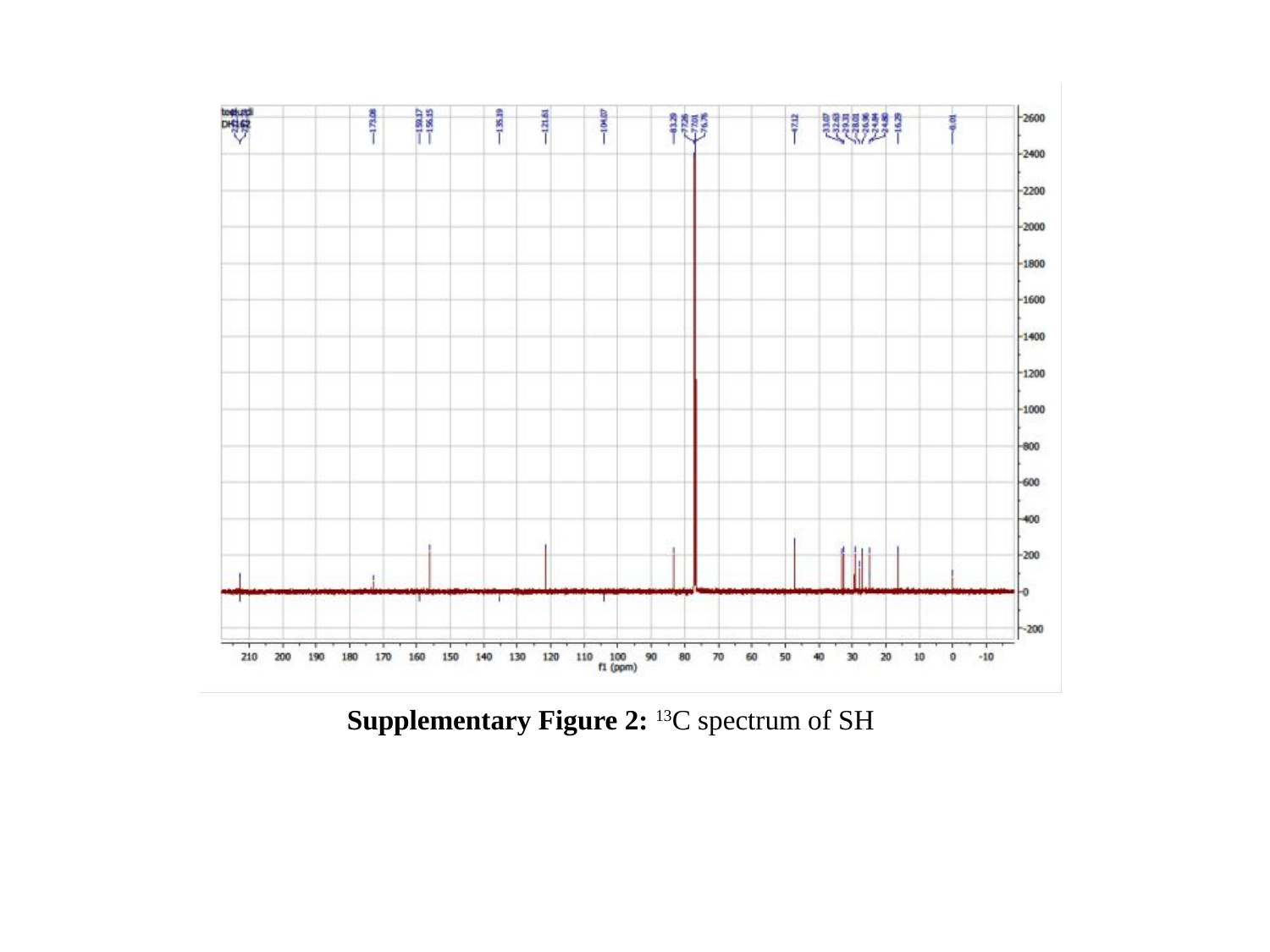

Supplementary Figure 2: 13C spectrum of SH

## Slide 3
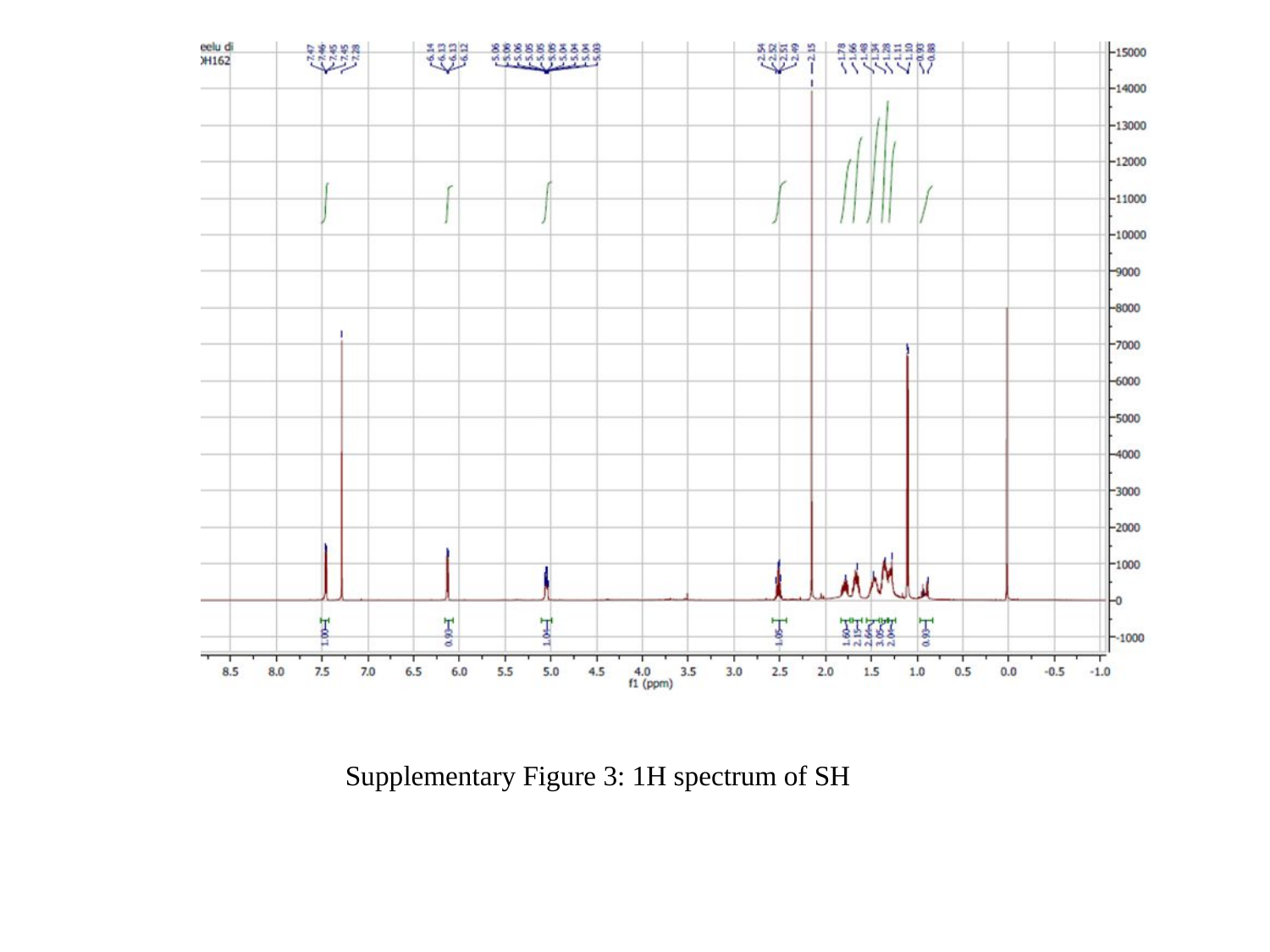

Supplementary Figure 3: 1H spectrum of SH

## Slide 4
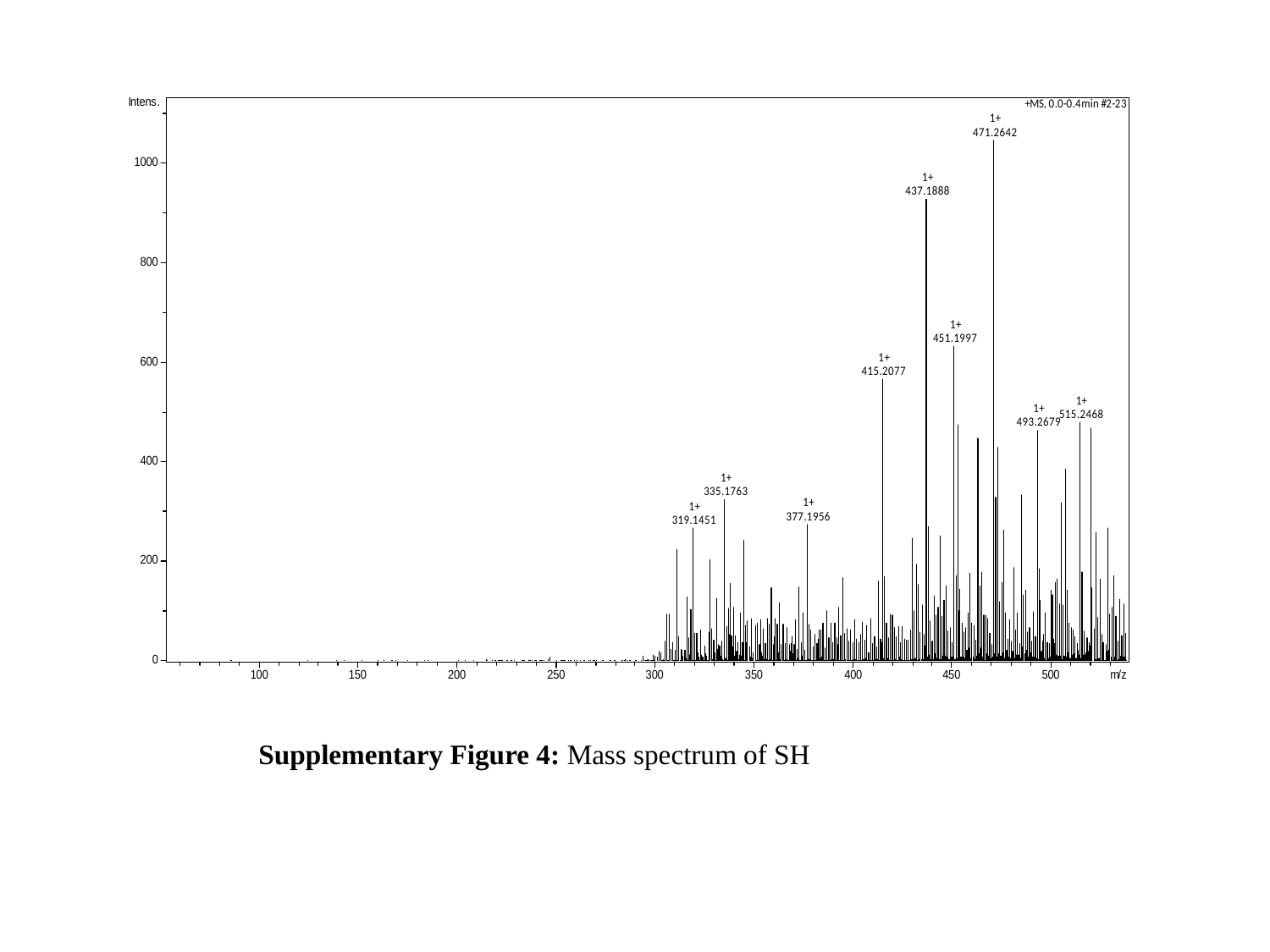

Supplementary Figure 4: Mass spectrum of SH
