# Supplementary figures and images for: Purification and Characterization of a New Antifungal Compound 10-(2,2-dimethyl-cyclohexyl)-6,9-dihydroxy-4,9-dimethyl-dec-2-enoic Acid Methyl Ester from Streptomyces hydrogenans Strain DH16
Source: Front Microbiol. 2016 Jun 29;7:1004. doi: 10.3389/fmicb.2016.01004 (PMC4926525; doi:10.3389/fmicb.2016.01004)

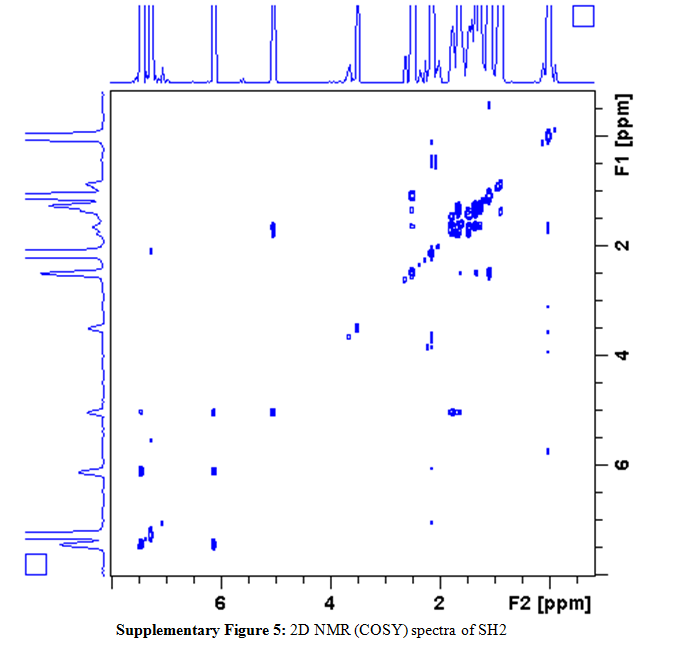

Supplement: Supplementary file 1 [file Presentation_1.ZIP › image 5.tif]
